# Clinical predictors of embryo quality among women of advanced age receiving intracytoplasmic sperm injection cycles in Malaysia: A cohort study

**DOI:** 10.18502/ijrm.v20i7.11560

**Published:** 2022-08-08

**Authors:** Ezanaton Nisar Omar Hafizi, Rahimah Abdul Rahim, Erinna Mohamad Zon, Adibah Ibrahim

**Affiliations:** ^1^Department of Obstetrics and Gynecology, Hospital Universiti Sains Malaysia, Health Campus, Kubang Kerian, Kelantan, Malaysia.; ^2^Department of Obstetrics and Gynecology, School of Medical Sciences, Health Campus, Universiti Sains Malaysia, Kubang Kerian, Kelantan, Malaysia.

**Keywords:** Embryo development, Intracytoplasmic sperm injection, Ovarian hyperstimulation, Advanced age, Predictors.

## Abstract

**Background:**

Declining fertility in a woman of advanced age is associated with a depletion in ovarian reserve as well as declining oocyte and embryo quality. Determining the predictors of embryo quality may assist in stimulation target and cycle prediction.

**Objective:**

This study aims to identify factors affecting embryo quality among women of advanced age receiving intracytoplasmic sperm injection (ICSI) cycles.

**Materials and Methods:**

This prospective cohort study was conducted over a period of 12 months, from January until December 2018, on 734 mature oocytes retrieved from 124 women of advanced age (35-45 yr old) receiving ICSI. The Society of Assisted Reproductive Techniques system was used to determine the morphological grading of embryo quality. The fertilization rate, cleavage rate, and pregnancy rate per cycle were expressed as a percentage per cycle for a total of 76 embryo transfers. Possible predictors of high-quality embryos were evaluated using single and multiple regression tests, with p < 0.05 considered as significant.

**Results:**

Out of the 586 available embryos, 288 (49.15%) high-quality embryos were obtained. The fertilization and cleavage rates were 86.18% and 97.83%, respectively. The total number of retrieved oocytes (R^2^ = 0.857) and the total available embryos (R^2^ = 0.857) were closely related to high-quality embryos. 76 embryo transfers were conducted, with 17 successful conceptions (implantation rate = 22.37% per transfer). There were no miscarriages among the pregnancies.

**Conclusion:**

Increasing the number of collected oocytes and the cleavage rate could increase the chance of obtaining more high-grade embryos. This could increase the success of ICSI among women of advanced age.

## 1. Introduction

Advanced maternal age (AMA), defined as the age of women that is 35-45 yr, is associated with a decline in both ovarian reserve and competency of oocyte and embryo (1-3). Among these women, the success rate of any fertility treatment is low (4, 5). The declining reproductive capacity among AMA women is associated with a depletion of oocytes in their ovaries. Several investigations have been proposed to evaluate ovarian reserve, which may be used to predict ovarian response to exogenous gonadotrophin stimuli (1, 6-13). The primary markers of ovarian reserve are age, basal follicle-stimulating hormone (FSH), anti-Mullerian hormone, and antral follicle count.

Besides the depletion in ovarian reserve, an increase in the rate of spontaneous miscarriage is also observed in AMA women, mainly because of oocyte abnormalities. The meiotic spindle in the oocytes of women with AMA frequently has abnormalities in chromosome alignment and microtubular matrix composition. A higher rate of aneuploidy has been observed among AMA women, which is the primary contributor to the increase in spontaneous miscarriage and reduced live birth rate (14, 15).

Despite these known age-related fertility issues, > 70% of patients attending our infertility center are women of advanced age. Many studies have been done to identify predictors of in vitro fertilization (IVF) outcome. A meta-analysis involving 14 studies showed a negative association between cycle outcome with female age and basal FSH, and a positive association with number of retrieved oocytes and quality of transferred embryo (16). Female age has been shown to be the strongest predictor. AMA affects not only the quantity of oocytes but also their quality and subsequently developed embryos. Embryo quality is one of the decisive factors of a successful IVF / intracytoplasmic sperm injection (ICSI) cycle. Presently, embryo quality is determined by its morphological appearance. Previous studies have identified multiple morphological kinetic markers that can predict embryo competence (1, 2). However, such identification requires technological competencies that may not readily be available in many centers, and in such centers, the use of clinical predictors of high-quality embryos would be especially beneficial in predicting the course of IVF cycles. In addition, these predictors can help physicians and couples to decide on the extent of treatment without unnecessarily increasing the cost. To date, predictors of embryo quality in women of advanced reproductive age are limited, and this is in contrast with data available on factors affecting live birth rates in these women.

The present study aimed to determine the clinical factors that contribute to the development of good-quality embryos among women of advanced age. We hypothesized that the level of FSH, the total dose of gonadotrophins used as ovarian hyperstimulation, and the number and quality of oocytes and embryos could predict the quality of embryos. This study may not be novel research. However, it may contribute to future meta-analyses or systemic review research on embryo quality.

## 2. Materials and Methods

This prospective, cohort study was conducted among women who received their first IVF/ICSI treatment. The present study was conducted over a period of 12 months, from January until December 2018.

### Inclusion and exclusion criteria

All women aged 35-45 yr who received their first IVF/ICSI treatment were recruited for the present study. Women with severe endometriosis, polycystic ovarian syndrome, uncontrolled preexisting diabetes mellitus, underlying psychiatric disorders, recurrent miscarriage, antiphospholipid syndrome, personal or family history of inherited congenital disorders, or a partner with an abnormal seminal fluid analysis were excluded. A total of 941 oocytes were retrieved for study analysis.

### Sample size calculation

**Figure 1:**
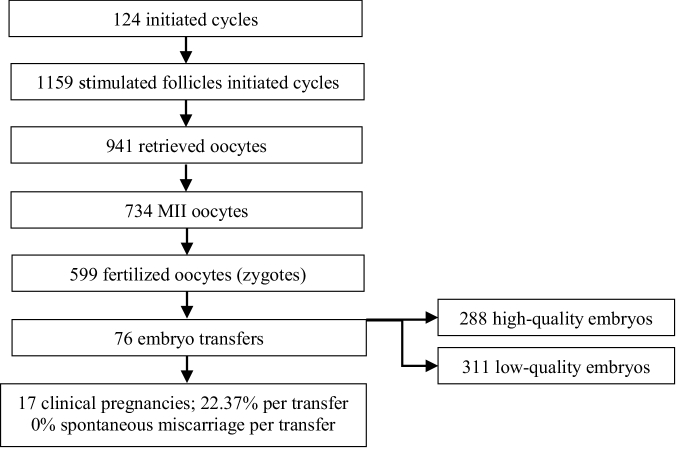
Study flow chart.

Using the estimation of the mean formula to obtain good-quality embryos, a minimum of 190 oocytes and 38 embryos were needed to meet the 95% confidence interval. We retrieved a total of 934 oocytes from 124 women, of which only 734 oocytes were metaphase II (MII) normal oocytes which had undergone the ICSI procedure (Figure 1).

### Ovarian stimulation and ICSI

All women in this study were stimulated with the short gonadotrophin-releasing hormone (GnRH) antagonist protocol. Ovarian hyperstimulation was initiated using recombinant gonadotrophin, follitrophin beta (PuregonⓇ, Organon Malaysia Sdn Bhd) and was dictated according to patients' responses to stimulation, which was evaluated by serial vaginal ultrasound examinations (Acuson 50, Siemen Healthineers, Erlangen, Germany). When the majority of the follicles reached 13-14 mm in diameter, GnRH antagonist, ganirelix (OrgalutronⓇ, Vetter Pharma-Pertigung GmbH & Co. KG) was added at a dose of 250 μg daily. Once at least 3 leading follicles reached a size of 18 mm, 10,000 IU intramuscular human chorionic gonadotrophin (hCG; PregnylⓇ, Merck Sharp & Dohme, Malaysia) was administered. During this time, the number of mature follicles, endometrial thickness, and level of serum progesterone were determined. Oocyte retrieval was then performed 34-36 hr after the hCG administration. Using the total oocyte scoring system, the oocytes were scored as -1 (poor quality), 0 (almost normal), and +1 (normal) oocytes (17). The semen was prepared using the density gradient method (18), and the most motile and normal sperm was selected for the ICSI procedure.

### Evaluation of fertilization, cleavage, and embryo grading

Fertilization was determined 17 hr after ICSI. The fertilized oocytes (zygotes) were further incubated and re-examined for embryo grading 48 hr later during which the embryo was graded based on morphology using the standardized morphological grading system of the Society of Assisted Reproductive Techniques (SART) committee, also referred to as the SARTCORS criteria, as described in table I (3). Based on these criteria, the embryos were graded as good-, fair-, or poor-quality embryos. To facilitate analysis, we grouped the fair-quality embryos with the good-quality embryos. Embryos were transferred on day 3 after oocyte retrieval. No more than 2 embryos were transferred at a time. Only good- and fair-quality embryos, up to a maximum of 2, were transferred in the present study. Embryos were expelled from the catheter, and the catheter was slowly withdrawn while maintaining a steady pressure on the plunger of the syringe. The catheter was then examined for complete expulsion confirmation. The luteal phase was supported with 100 mg of micronized progesterone vaginal gel (CrinoneⓇ progesterone gel 8%, Merck Sdn Bhd), which was administered daily, and 10 mg of oral progestogen taken twice a day.

### Pregnancy confirmation

3 wk after the embryo transfer, 3 cc of venous blood was drawn to determine the level of beta human chorionic gonadotropin. A beta human chorionic gonadotropin of > 0.2 IU/L was considered a successful IVF cycle and was taken as a biochemical pregnancy. An ultrasound scan of the uterus was conducted 2 wk later to determine the presence of a gestational sac, which was considered a successful clinical pregnancy. Only successful clinical pregnancies were considered for analysis.

**Table 1:** SARTCORS criteria for embryo grading


**Growth phase**		**Cell criteria**
**Cleavage**		
	**Good**	Fragmentation: 0- < 10%, inner cell mass: perfectly symmetrical
	**Fair**	Fragmentation: 11-25%, inner cell mass: moderately asymmetrical
	**Poor**	Fragmentation: > 25%, inner cell mass: severely asymmetrical
**Morula**		
	**Good**	Fragmentation: 0- < 10%, inner cell mass: complete or incomplete compaction
	**Fair**	Fragmentation: 11-25%, inner cell mass: incomplete compaction
	**Poor**	Fragmentation: 11-25%, inner cell mass: incomplete compaction

### Ethical considerations

The study protocol was approved by the local Human Medical Research and Ethics Committee, Kelantan, Malaysia (Code: USM/JEPeM/16020055), which has adopted the ethical guidelines outlined by the Declaration of Helsinki. Written consent was obtained from all participants prior to study recruitment.

### Statistical analysis

The Statistical Package for the Social Sciences (IBM SPSS Statistics for Windows, version 24.0, IBM Corp, Armonk, NY, USA, released 2017) was used for data analysis. Study population and cycle characteristics were analyzed using descriptive statistics, in which numerical data were presented as mean and standard deviation, whereas categorical data were presented as a percentage. The association between age, duration of subfertility, body mass index (BMI), baseline FSH and luteinizing hormone (LH) levels, the total dose of gonadotrophin used, and the number of stimulated oocytes, mature oocytes, and total available embryos, with high-quality embryos, were analyzed using a simple linear regression test and multiple linear regression test. A p-value of < 0.05 was considered statistically significant.

The fertilization rate per cycle was determined as the percentage of number of zygotes over the total oocytes that underwent ICSI. The cleavage rate per cycle was determined as the percentage of cleaved embryos over the total zygotes, and the pregnancy rate per transfer was determined as the percentage of successful clinical pregnancies over the number of transfers.

## 3. Results

### Patient characteristics

A total of 941 stimulated oocytes were retrieved, of which 734 were mature and used for ICSI. The fertilization rate per cycle was 86.18%, and the cleavage rate per cycle was 97.83%. Out of the 586 available embryos, 288 (49.15%) were high-quality, while the rest were low-quality embryos (n = 298, 50.85%).

The implantation rate was 22.37%. There were no cases of miscarriage or ectopic pregnancy, resulting in a clinical pregnancy rate identical to the implantation rate of 22.37% per cycle. The baseline characteristics of the study population are summarized in table II. The mean maternal age of women undergoing ICSI treatment was 38.21 ± 2.78 yr. The average duration of subfertility was 7.43 ± 4.09 months. The mean BMI, baseline FSH, and baseline LH were 24.99 ± 4.75 kg/m^2^, 5.35 ± 2.50 mIU/m, and 5.18 ± 3.08 mIU/m, respectively. No significant difference was noted between the study characteristics and embryo quality groups, either high- or low-quality embryos.

### Cycle characteristics and response to stimulation

All patients received the same starting dose of gonadotrophin, 300 IU daily, for stimulation. The average dose of gonadotrophin used was 2270.97 ± 798.90 IU. A total of 1159 follicles were stimulated, with a mean of 9.35 ± 4.84 per cycle, of which a mean of 7.59 ± 5.12 oocytes per cycle were retrieved. The endometrial thickness on hCG administration day, embryo quality, and fertilization, implantation, and clinical pregnancy rates are displayed in table III. The total dose of gonadotrophin used, size of the stimulated follicles, duration of stimulation, endometrial thickness, and level of progesterone on the day of hCG administration were comparable between the 2 groups (Table III).

### Predictors of high-quality embryos

A correlation coefficient test as presented in table IV showed good correlations between the total stimulated follicles, retrieved oocytes, mature oocytes, high-quality oocytes, fertilized oocytes, and the obtained high-quality embryos (r = 0.297, 0.579, 0.634, 0.759, 0.908, and 0.917, respectively).

The single linear regression, which showed that the baseline LH, number of stimulated follicles, number of retrieved oocytes, number of mature oocytes retrieved, number of zygotes, and total number of available embryos could predict the development of high-quality embryos (p = 0.016, 0.001, < 0.001, 0.001, < 0.001, and < 0.001, respectively), is presented in table V. However, in the multiple linear regression analysis, only the number of retrieved oocytes and the number of available embryos could independently make such a prediction (Table V). 8 high-quality embryos were predicted to be produced for every 10 retrieved oocytes (p < 0.001). The number of mature oocytes could not significantly predict high-quality embryos. For every 10 available embryos, 9 were predicted to be of high quality (p < 0.001).

**Table 2:** Descriptive statistics of the study population


**Characteristic**		**Estimates of study population**
Maternal age (yr)*		38.21 ± 2.78
BMI (kg/m^2^)*		24.99 ± 4.75
Type of infertility**		
	**Primary**	102 (82.26)
	**Secondary**	22 (17.74)
Duration of infertility (yr)*		7.43 ± 4.09
Baseline FSH (mIU/mL)*		5.35 ± 2.50
Baseline LH (mIU/mL)*		5.18 ± 3.08
*Data presented as Mean ± Standard deviation. **Data presented as n (%). BMI: Body mass index, FSH: Follicle-stimulating hormone, LH: Luteinizing hormone		

**Table 3:** Cycle characteristics and stimulation response


**Characteristic**	**n (%)**	**Mean ± SD**
**Gonadotrophin dose used per cycle (IU)**	- 2270.97 ± 798.90
**Duration of stimulation (days)**	- 10.10 ± 1.90
**Stimulated follicles number**	1159	9.35 ± 4.84
**Stimulated follicles size (mm) **	- 14.86 ± 2.16
**Endometrial thickness (mm)**	- 9.89 ± 2.52
**Serum progesterone on the day of hCG administration (nmol/L)**	- 3.98 ± 3.13
**Oocytes retrieved per cycle**	941	7.59 ± 5.12
**Good-quality oocytes**	563	4.55 ± 3.13
**Poor-quality oocytes**	171	1.52 ± 0.92
**Mature oocytes retrieved per cycle**	734	5.60 ± 3.73
**Zygote available (fertilized oocytes)**	599 (86.18)*	
**Available embryos **	586 (97.83)**	
**High-quality embryos**	288 (49.15)	
**Embryo transfer**	76	
**Implantations**	17 (22.37)***	
SD: Standard deviation, hCG: Human chorionic gonadotrophin. The percentage of retrieved oocytes was calculated by taking the percentage of the number of retrieved oocytes over the number of stimulated follicles. *Fertilization rate per cycle, **Cleavage rate per cycle, ***Implantation rate per transfer		

**Table 4:** Correlation between various cycle characteristics with high-quality embryos


	**Number of high-quality embryos**	
**Variables**	**r**	**P-value**
	**Age (yr)**	0.093	0.305
**Infertility duration (yr)**	0.020	0.823
**BMI (kg/m²)**	0.071	0.436
**FSH baseline (miu/ml)**	-0.085	0.347
**LH baseline (miu/ml)**	0.215	0.016
**Total dosage of gonadotrophin (FSH) stimulation (IU)**	-0.002	0.985
**Duration of gonadotrophin (FSH) stimulation (days)**	-0.124	0.171
**Number of total follicles**	0.297	0.001
**Size of follicles**	-0.006	0.949
**Endometrium thickness (mm)**	0.051	0.573
**Progesterone on hCG day (nmol/L)**	0.018	0.847
**Total number of oocyte retrieved per cycle**	0.579	< 0.001
**Number of mature (MII) oocytes retrieved per cycle**	0.634	< 0.001
**Number of good-quality oocytes retrieved per cycle**	0.759	< 0.001
**Number of fertilized oocytes per cycle**	0.908	< 0.001
**Number of embryos obtained per cycle**	0.917	< 0.001
r = Correlation coefficient, BMI: Body mass index, FSH: Follicle stimulating hormone, LH: Luteinizing hormone, MII: Metaphase 2		

**Table 5:** Linear regression test for predictors of high-quality embryos


	**Simple linear regression test ${}^{\text{a}}$ **			**Multivariable regression test ${}^{\text{b}}$ **	
**Variables**	**Crude b (95% CI)**	**R²**	**P-value**	**Adjusted b (95% CI)**	**P-value**
	**Age (yr)**	0.102 (-0.094, 0.299)	0.009	0.305	- -
**Duration of infertility (yr)**	0.015 (-0.119, 0.149)	0.000	0.823	- -
**BMI (kg/m²)**	0.046 (-0.070, 0.161)	0.005	0.438	- -
**FSH baseline (mIU/mL)**	-0.104 (-0.323, 0.114)	0.007	0.347	- -
**LH baseline (mIU/mL)**	0.214 (0.040, 0.387)	0.046	0.016	0.032 (-0.042, 0.106)	0.389
**Total dosage of FSH stimulation (IU)**	-0.000006 (-0.001, 0.001)	0.000	0.985	- -
**Duration of stimulation (days)**	-0.200 (-0.486, 0.087)	0.015	0.171	- -
**Follicle number**	0.188 (0.080, 0.296)	0.088	0.001	-0.026 (-0.086, 0.034)	0.396
**Follicle size (mm)**	-0.008 (-0.262, 0.245)	0.000	0.949	- -
**Endometrium thickness (mm)**	0.062 (-0.155, 0.279)	0.003	0.573	- -
**Progesterone on hCG day (nmol/L)**	0.017 (-0.158, 0.192)	0.000	0.847	- -
**Total oocytes retrieved**	0.346 (0.259, 0.434)	0.336	< 0.001	0.847 (-0.135, -0.017)	< 0.001
**Mature oocytes retrieved (MII)**	0.520 (0.406, 0.634)	0.401	< 0.001	0.024 (-0.082, 0.130)	0.653
**Good-quality oocytes retrieved**	0.742 (0.628, 0.856)	0.576	< 0.001	0.118 (0.005, 0.231)	0.041
**Fertilized oocytes**	0.842 (0.772, 0.912)	0.824	< 0.001	0.15 (-0.220, 0.510)	0.434
**Total embryos obtained**	0.871 (0.803, 0.938)	0.841	< 0.001	0.870 (0.748, 0.993)	< 0.001
${}^{\text{a}}$ Simple linear regression analysis was used to find possible predictors, significant predictors were further tested using multivariable regression test, R^2^ = 0.857 indicates a strong correlation between tested variables with high-quality embryos. ${}^{\text{b}}$ Multivariable regression test was used to find predictors of high-quality embryos, R^2^ = 0.857 indicates a strong linear correlation. BMI: Body mass index, FSH: Follicle-stimulating hormone, LH: Luteinizing hormone, hCG: Human chorionic gonadotrophin, MII: Metaphase 2					

## 4. Discussion

Women's age has been identified as a strong independent factor for the outcome of IVF. By the age of 35-39 yr, the rates of spontaneous cumulative pregnancy start to decline, and they approach almost zero immediately after 45 yr (1, 4, 6, 7, 19). The reason for this trend is believed to be the gradual depletion in the ovarian reserve associated with a progressive decrease in oocytes and embryo competence, which has become a great challenge for fertility physicians. In the present study, we evaluated the potential factors that could predict the quality of embryos among women of advanced age.

Currently, multiple systems to grade and rank embryos are available and these range from a simple grading system that assigns a single grade to account for all aspects of post-cleavage embryo appearance to more complex systems that use a formula to predict the likelihood of pregnancy based on embryo appearance and development (6, 8, 9, 19). In the present study, we used the SARTCORS grading system developed by the SART committee, which classifies embryos on a 3-point grading system (good, fair, and poor) based on their morphology (3).

At the beginning of the study, we hypothesized that embryo quality is affected by the level of FSH, the total dose of gonadotrophins used as ovarian hyperstimulation, the number and quality of oocytes, and the number of available embryos. In this study, although AMA was associated with reduced ovarian reserve, neither the FSH level nor the dosage of gonadotrophin used as ovarian stimulation could predict a high-quality embryo.

The present study demonstrated that embryo quality was affected by the number of retrieved oocytes and the number of available embryos. One could expect to have 8 high-quality embryos for every 10 retrieved oocytes. The number of retrieved oocytes decreased across various age groups and was highest among women younger than 30 yr (mean 18.5 ± 10.3 yr) and decreased significantly to 9.8 ± 6.7 for those 39 yr old and to 4.5 ± 2.3 for those older than 45 yr. In the present study, at a mean age of 38.21 ± 2.78 yr, the mean retrieved oocytes per cycle was 7.59 ± 5.12, which was about the expected rate.

Previous studies have indicated that age is predictive not only for the number of oocytes but also the number of MII oocytes, oocyte quality, and embryo quality (14-20). It was somewhat surprising to note that we retrieved more good-quality oocytes (grade 1; mean 4.55 ± 3.13) than poor-quality oocytes (grades 2 and 3; mean 1.38 ± 2.82) from the population of the present study. This could be because most of our patients were younger than 40 yr of age (mean 38.21 ± 2.78 yr). However, this study has demonstrated a significant association between good-quality oocytes and high-quality embryos as predicted.

In contrast to results reported previously (9, 11, 21, 22), we show that embryo quality was not affected by the number of MII oocytes, after stratification by multivariable regression test (r^2^ = 0.401, p = 0.653). We standardized the type and dosage of ovarian stimulating agents and embryo culture media and environment for our study population to make statistical analysis easier. These factors could have contributed to the divergent findings of the present study.

Many studies have tried to identify ways of increasing the number of stimulated oocytes from AMA patients (12-16, 21-23). In his review of ovarian stimulation regimens for poor responders to IVF, Blumenfeld stated that the best treatment for these patients is egg donation (20); however, this method cannot be universally applied. In contrast, Huang et al. suggested the use of the GnRH antagonist protocol in older poor responders and the GnRH agonist protocol in younger patients (24). Various other regimens have been proposed to improve ovarian response in poor responders, including the addition of LH (23, 25), androgen (15, 23, 25), and growth hormone (20, 26-28) to exogenous gonadotrophin during stimulation.

Increasing the number of available embryos could also increase the chance of obtaining a good-quality embryo, and embryo numbers can be improved by increasing the number of retrieved oocytes and by improving culture techniques. Much effort has been directed in improving culture techniques, including refining culture media and using insemination before the availability of time-lapsed incubators to minimize the temperature gradient during monitoring. Farhi and colleagues reported a significant improvement in embryo quality when ICSI was implemented instead of IVF among AMA (7).

## 5. Conclusion

There is evidence that the embryo quality declines with maternal age. This study showed that the chance of obtaining a high-quality embryo can be improved by increasing the number and quality of collected oocytes and the number of available embryos. This measure can be achieved by regulating the stimulation regimens and methods of embryo incubation.

##  Conflict of Interest

The authors declare that there is no conflict of interest.
